# Baron Liebig’s Soup for Children

**Published:** 1865

**Authors:** 


Baron Liebig's Soup for Children.—With that remarkable estimation of the greatness of small things which is one of the most valuable of his many high intellectual qualities, and with a tender appreciation of the importance of small people, Baron Liebig devotes a special article in an English scientific periodical to the description of a new article of diet which he conceives to be the most fitting substitute for the natural nutriment for those children who are by circumstances robbed of their mother's milk. It is well known that cow's milk does not adequately represent the milk of a healthy woman, and when wheaten flour is added, as it commonly is, Liebig points out that, although starch be not unfitting for the nourishment of the infant, the change of it into sugar in the stomach during digestion imposes an unnecessary labor on the organization, which will be spared it if the starch be beforehand transformed into the soluble forms of sugar and dextrine. This he effects by adding to the wheaten flour a certain quantity of malt. As wheaten flour and malt flour contain less alkali than woman's milk, he supplies this when preparing the soup. This "soup" may be shortly prepared as follows:—"Half an ounce of wheaten flour, and an equal quantity of malt flour, seven grains and a quarter of bicarbonate of potash, and one ounce of water, are to be well mixed; five ounces of cow's milk are then to be added, and the whole put on a gentle fire; when the mixture begins to thicken it is removed from the fire, stirred during five minutes, heated and stirred again till it becomes quite fluid, and finally made to boil. After the separation of the bran by a sieve, it is ready for use. By boiling it for a few minutes it loses all taste of the flour.

The immediate inducement for his making the soup was that one of his grand-children could not be suckled by its mother, and that another required, besides his mother's milk, a more concentrated food. In both cases, as well as in other families where it had been introduced, the soup proved an excellent food, the children thrived perfectly well, and many a petty suffering disappeared after some weeks' use of the soup. He often takes it prepared with ten parts of milk and two parts of malt flour, with tea, for his breakfast. He adds that "Dr. Von Pleufer, the most renowned physician in Munich, has induced the apothecaries of the town to keep for sale a mixture of half an ounce of malt flour and seven grains and a quarter of bicarbonate of potash, milk and wheat flour being supposed to be in every house. The malt flour ought to be always freshly made from the malt.—*Lancet*, Jan. 7.

